# Percentage on Prescriptions

**Published:** 1871-01

**Authors:** 


### Percentage on Prescriptions.

The custom of allowing to physicians a *per centum* on prescriptions, so common many years since, we had supposed entirely abandoned; but a communication received by a respectable practitioner of Buffalo contains the following sentence:

"We therefore say, should we meet your confidence, all prescriptions you may send us, we will allow you 10 per cent. of the amount of sales. Patrons bringing one of the enclosed sheets, the same will be considered as coming from you, when containing your endorsement, and will be filed to your credit, and per centage paid quarterly.

"Very Respectfully, ———."

We mistrust that few physicians are small enough, and sordid enough to sponge, in this detestable way, an additional six-pence from patients; and we wonder that even one dealer in medicinal substances could be so misguided as to offer any such imposition to a physician. The note prefaces very properly, "should we meet your confidence:" certainly no druggist will meet the confidence of any honorable physician who makes such propositions. Physicians are expected to hold their good names, their reputation and honesty at a much higher rate than 10 *per centum* on the amount of such dishonorable sales.

